# Impact of cancelling foundation year rotations due to the COVID-19 outbreak in the UK

**DOI:** 10.1136/postgradmedj-2020-137775

**Published:** 2020-04-19

**Authors:** Anni Ding, Yuanpei Zhang

**Affiliations:** Academic Foundation Doctor, Imperial College Healthcare NHS Trust, London, UK; Academic Foundation Doctor, Torbay and South Devon NHS Foundation Trust, Torquay, Torbay, UK

**Keywords:** medical education & training, infectious diseases, public health

We, like many other doctors around the country, have been following the COVID-19 outbreak in the UK with great interest.

Coronaviruses (CoV) are a family of viruses that can cause a spectrum of respiratory illnesses, which range from the common cold, to more severe diseases such as severe acute respiratory syndrome (SARS-CoV) and Middle East respiratory syndrome (MERS-CoV). COVID-19 is a respiratory disease caused by a novel coronavirus, SARS-CoV-2. The disease first emerged in December 2019, associated with a seafood market in Wuhan, China. Since then, it has become pandemic, affecting at least 176 countries and territories worldwide. At the time of writing, there have been 803 650 cases and 39 033 deaths globally. The number of cases in the UK has been rising dramatically; in total, 22 465 cases of COVID-19 has been identified with 1412 deaths.^1^ The scale of the public health crisis posed by COVID-19 has been unparalleled in recent times, putting the National Health Service (NHS) under extreme stress, particularly in London. Indeed, the ExCeL centre in London is currently being converted into a makeshift hospital with a maximum capacity of 4000 beds in order to cope with the rapidly increasing number of COVID-19 cases in the capital.

There are approximately 15 000 foundation doctors in the UK. These doctors rotate through a mixture of predetermined medical and surgical specialties every 4 months over a period of 2 years after graduation from medical school.^2  3^ The rotations enable the doctors to gain a range of experiences across different specialties, which is important for a multitude of factors; it helps to build a key set of core knowledge and skills, allows the doctor to identify the specialties to which they are suited to, and prepares them for the next stage of their postgraduate training.

Higher Education England (HEE) decided in mid-March to cancel all rotations that were due to take place in April 2020, unless local deaneries make specific arrangements otherwise.^4^ This is due to foreseen pressure on the NHS from the ever-growing COVID-19 outbreak in the UK. In addition, HEE has acknowledged that some trainees may be redeployed to specialties expected to have a high volume of patients, namely the Accident and Emergency Department, and acute medical specialties in order to better distribute pressures and support their colleagues already working in those specialties. The decision has drawn mixed responses from the foundation year (FY) doctors; while almost all appreciate that this is an unprecedented time for the NHS that requires coherence as a community, it has been nevertheless disappointing news to significant proportion of doctors. Many were welcoming to a change of scenery after 4 months, or looking forward to a rotation that they have a particular interest in. For the Academic Foundation Trainees due to their research block, their hopes of flexible working hours and a break from clinical work has been dampened, with all academic rotations cancelled. From hereon, the authors present the advantages or disadvantages of such a decision from HEE.

There are a number of positives associated with halting FY rotations. Remaining in the same post bypasses the need for departmental induction, which otherwise would be customary at the start of each rotation. Moreover, it removes issues associated with unfamiliarity and the need to adjust to a new post. This helps to maintain the level of patient care and minimise disruptions, while maximising resource management at a time that the NHS resources are being severely stretched. A systematic review of 14 studies conducted in the UK found that doctors were at high risk of burnout, with 31%–54% of doctors feeling emotionally exhausted.^5^ Emotional and psychological support at this time is crucial in alleviating stress and navigating the unprecedented demands of work. Anecdotally, doctors, including senior consultants are struggling to deal with the emotional impact of seeing extremely sick COVID-19-infected patients. Many doctors are working beyond the normal contracted hours, this combined with social distancing away from work can make doctors extremely vulnerable to mental health issues. By working with colleagues that they have already built a rapport with, FY doctors will likely be better supported during this demanding time, and likewise, be better placed to reciprocate this support. Indeed, the emotional strain placed on all NHS workers are being recognised, with hospital trusts offering counselling service, advice helplines and apps such as Headspace and Unmind offering free subscription to NHS staff to help look after their mental well-being.

To a proportion of doctors, the prospect of working another 4 months in the current specialty could be disappointing and anxiety-inducing. The last rotation of the year may have been the one they have been looking forward to, or may be the specialty they wish to pursue, and they now miss out on the opportunity to experience that specialty. The purpose of the Foundation Programme is to give junior doctors a wide range of clinical experience and the pandemic may adversely affect the breadth of experience gained. For many doctors, they may not be enjoying their current placement, and were beginning to find the work monotonous after nearly 4 months and were looking forward to starting afresh in a new clinical setting. This can be especially true for more vigorous rotations with longer hours or more frequent on calls. The true psychological impact of cancellation of rotations is difficult to estimate, and the impact due to lack of rotation itself may be challenging to separate from the stresses of working under an increasingly pressured NHS and managing an increasing number of COVID-19 cases. A prospective or retrospective study on the psychological impact of COVID-19 on doctors would be interesting in order to assess this. Job satisfaction has been shown to be protective against the effect of stress on emotional exhaustion; increasing job stress and work overload are associated with psychiatric morbidity.^3^

The COVID-19 pandemic is and will be an extraordinary challenge for the NHS and profoundly impact all its associated staff. It is inevitable that all doctors will be forced to deal with novel situations and take on roles beyond the scope of their normal work. FY doctors are a fundamental part of the NHS response to this unprecedented crisis. While there may be mixed emotions towards the decision by the HEE to cancel the last rotation of the academic year, the main priority is on patient care and safety, which requires a united front by all healthcare workers. The question of whether the last rotation of the year can take place later remains to be seen and is dependent on the trajectory of the disease spread in this country.
